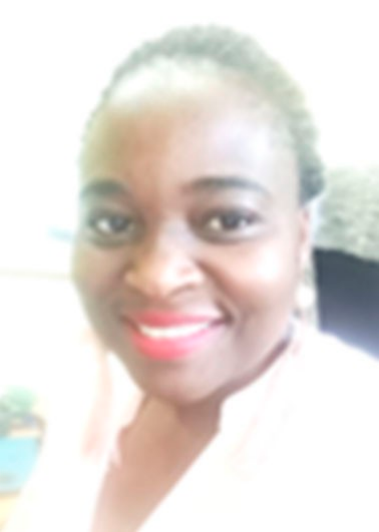# Activity of Riminophenazines against *Mycobacterium tuberculosis*: A Review of Studies that Might be Contenders for Use as Antituberculosis Agents

**DOI:** 10.1002/cmdc.202000580

**Published:** 2020-10-15

**Authors:** Mpelegeng Victoria Bvumbi

**Affiliations:** ^1^ Department of Chemistry University of Venda Private Bag X5050 Thohoyandou 0950 South Africa

**Keywords:** drug discovery, medicinal chemistry, MDR-TB, *Mycobacterium tuberculosis*, riminophenazines

## Abstract

Tuberculosis is one of the leading cause of death in the world, mainly due to the increasing number of multidrug‐resistant TB (MDR‐TB) and extensively drug‐resistant TB (XDR‐TB) strains. Factors such as the HIV pandemic contribute further. Also, the ineffectiveness of the chemotherapy in current use increases the mortality rate. Therefore, new and repurposed antituberculosis drugs are urgently needed for the treatment of MDR‐TB, and riminophenazines are among those drugs that are being reinvestigated for their potential in the treatment of TB. This review delivers a brief historical account of riminophenazines, their general synthesis, mechanisms of action, and their physicochemical properties. The discussion is limited to those studies that investigated the activity of these compounds as antituberculosis agents. Given their unique properties, this review will be of great significance in giving direction towards the design and development of new riminophenazine analogues.

## Graphical abstract

**Riminophenazines innovation**: Substituents on the phenazine nucleus afford novel molecules with improved antituberculosis potency. This review outlines developments in the riminophenazine analogues and summarizes synthesis, mechanism of action and physicochemical properties.

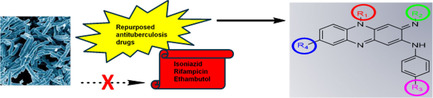

## 1 Introduction

Tuberculosis (TB) is an infectious disease that is caused by the bacterium *Mycobacterium tuberculosis* and it continues to be a serious threat to public health.^[1]^ It is ranked above HIV/AIDS as the single infectious agent that causes the most deaths worldwide. While approximately one‐third of the world's population is infected with latent TB,^[2]^ in 2016 an estimated 10.4 million people developed active TB resulting in 1.67 million deaths.^[3]^ Moreover, hundreds of thousands (480 000) of multi‐drug‐resistant tuberculosis (MDR‐TB) cases and 190 000 deaths are estimated to have occurred in 2014.^[4]^ Recently, the disease has been on the increase, mainly because of HIV co‐infections.[^4^, ^5^, ^6^, ^7^, ^8^] Furthermore, diabetes, poverty, alcohol abuse, malnutrition and tobacco smoke are also considered contributory factors associated with the rise in TB infections.[^5^, ^9^, ^10^, ^11^, ^12^]

To date, there is a great challenge for the control and treatment of TB. This is due to the increased number of MDR‐TB, resistant to first‐line drugs such as isoniazid, rifampicin and ethambutol,[^13^, ^14^]) and extensively drug‐resistant TB (XDR‐TB).[^15^, ^16^, ^17^, ^18^] Furthermore, only one in three cases of MDR‐TB is diagnosed globally, with only one in four offered treatment for the disease.^[19]^ Therefore, new drugs as well as repurposed antituberculosis drugs, are needed urgently for the treatment of MDR‐TB in safer and more effective ways.[^20^, ^21^] Some well‐known repurposed agents include β‐lactams, clarithromycin, fluoroquinolones and linezolid.

**Figure 1:**
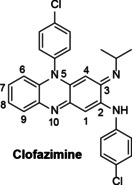
Structure of clofazimine.

There is a class of tricyclic heterocycles which has a phenazine ring substituted on one of the ring nitrogens forming the central phenazine ring. These are called riminophenazines and have shown various properties on different diseases. The name or designation “rimino” was coined to indicate the “R” substituent on the imino moiety (C‐3) of the molecule.^[22]^ These compounds were first derived from lichens and were developed many decades ago as TB drugs.[^23^, ^24^, ^25^, ^26^] Barry et al. initially discovered a compound called anilinoaposafranine[^23^, ^24^, ^27^] and it was active against tubercle bacilli. Following this discovery, they further prepared several structural modifications to the (NH) imino group, hence the name “rimino” group[^23^, ^24^, ^25^, ^27^, ^28^] Among the compounds which were prepared was a compound called B663, which was later named clofazimine (Figure 1).

This drug, along with its analogues, proved to be active and also accumulative inside the cells of the mononuclear phagocyte system. The drug was, however, delayed for use in the treatment of TB because it was not active in animal models like guinea pigs and monkeys.^[26]^ Clofazimine has been used for the treatment of leprosy since 1969, hence today it is regarded as a “repurposed” agent for TB.^[19]^ Furthermore, the drug is currently used as a WHO group‐five drug for MDR‐TB – meaning that it is not recommended for routine use for treatment of MDR‐tuberculosis because of unclear efficacy[^29^, ^30^] The action of clofazimine was hypothesized by Barry et al.^[23]^ and also supported by Yano et al.^[31]^ where they proposed intracellular redox cycling mechanisms as the basis for the activity, in effect functioning as redox “traps” that deplete cells of FADH and/or NAD(P)H.

Although there is a considerable body of literature on the activity of riminophenazine compounds on other mycobacteria such as *Mycobacterium leprae*, *Mycobacterium avium* complex (MAC) and *Mycobacterium smegmatis*,[^32^, ^33^, ^34^, ^35^, ^36^, ^37^, ^38^, ^39^, ^40^] in this review, the discussion of these compounds is limited only to the treatment of or activity on tuberculosis. Also, a summary of the synthesis, mechanism of action and physicochemical characteristics of this class of compounds are discussed.

## 2 New Anti‐TB Drug Development in Riminophenazines

A major goal of many anti‐TB drug discovery programmes is to develop treatments that will require a short duration. Hence the development of riminophenazine analogues which have proved to address the issue.

### 2.1 Chemistry (synthesis)

**Scheme 1:**
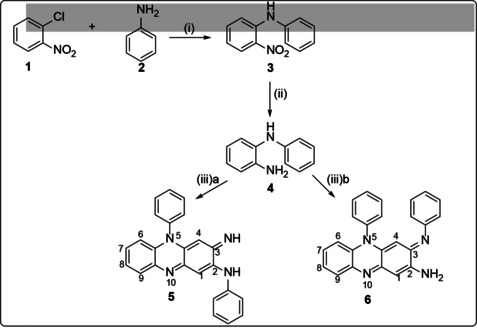
i) Pyridine, 200 °C; ii) EtOH, Raney nickel or Adams catalyst, iii‐a) FeCl_3_, HCl; iii‐b) *p*‐benzoquinone.

Generally, there are two methods used for the preparation of riminophenazine compounds. In the first method, Barry and co‐workers discovered that the condensation of two molecules of N‐chlorophenyl‐1,2‐diaminobenzene by oxidation with FeCl_3_ to form the tricyclic core of a riminophenazine gives the same substituents on the 2‐N‐phenyl and 5‐phenyl rings (Scheme 1).[^25^, ^41^] The synthesis proceeded as follows: Firstly, nucleophilic aromatic ipso‐substitution of the chloro group of the highly activated *o*‐chloronitrobenzene **1** by aniline **2** in the presence of pyridine took place, to give diphenylamine **3**. The o‐nitro group of **3** was then reduced by catalytic hydrogenation using Raney nickel or Adams catalyst (PtO_2_^.^H_2_O) in aqueous alcohol and in the presence of a small quantity of acetic acid to afford N monosubstituted *o*‐phenylenediamine **4**. Oxidative heterodimerisation was accomplished using either Fe^3+^ or benzoquinone as oxidant, presumably through the intermediacy of an o‐quinonediimine. Two regioisomers, **5** and **6** respectively, were obtained, with **6** being the major product in the benzoquinone oxidation, while **5** was present in a greater quantity in the ferric chloride route.

**Scheme 2:**
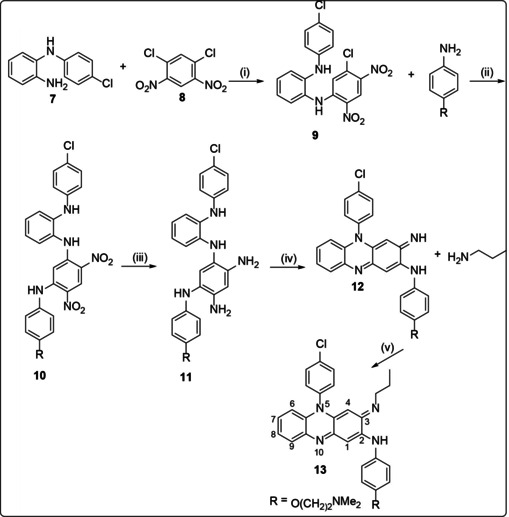
Girard and Ray's route to “asymmetrical” riminophenazines. i) EtOH, Et_3_N, reflux, 1 h; ii) isopropanol, reflux; iii) acetic acid, zinc powder, 20–40 °C; iv) air or O_2_; v) acetic acid, reflux, 5 h.

In order to allow different substituents at the C‐2 and N‐5 positions in the phenazine skeleton, Girard and Ray,^[42]^ used a step‐by‐step introduction of different substituted anilines onto the phenazine scaffold (Scheme 2). The first step involved the condensation of 1,3‐dichloro‐4,6‐dinitrobenzene **8** with 2‐amino‐4′‐chlorodiphenylamine **7** in ethanol, in the presence of a base to afford the ipso‐substituted dinitro compound **9**. Nucleophilic substitution of the remaining chloro group in **9** with 4‐(2‐dimethylaminoethoxy) aniline in isopropanol gave the aromatic dinitro phenyl as a key intermediate **10**. The nitro groups were then reduced under Clemmensen conditions (acetic acid and zinc powder at 20–40 °C) to give **11**. Aerobic oxidation of **11** took place either upon prolonged agitation in open air or by bubbling pure oxygen into the filtrate (after the zinc acetate and the excess metal were separated by filtering). After oxidation had occurred, the solution was basified with concentrated ammonia and dried to give the crude imino compound **12** as a highly coloured (red) resin. An excess of propylamine in the presence of acetic acid was added to this crude product which, upon heating, produced riminophenazine **13**, isolated by precipitation with an 85 % yield.

### 2.2 Mechanism of action

**Scheme 3:**
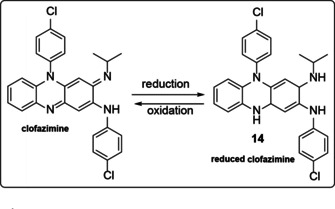
Redox ability of clofazimine.

Barry et al. who discovered clofazimine suggested that its antimycobacterial activity was attributable to two properties, namely clofazimine's high lipophilicity and redox potential.^[23]^ These were C log P=7.50 and a redox potential of −0.18 V at pH 7.0 respectively. They proposed that intracellular redox cycling was the likely mechanism of action of clofazimine. This requires the oxidation of reduced clofazimine, leading to the generation of the reactive oxygen species implicated in oxidative stress, such as superoxide and peroxide. It was supported by their finding that when clofazimine was incubated with the *M. tuberculosis* H_37_Rv strain under aerobic conditions, the culture turned a similar red to the compound. On the other hand, under anaerobic conditions, the culture became colourless **14**, indicating a loss of molecular conjugation due to reduction (Scheme 3).

In addition to their antimycobacterial activity, riminophenazines also exhibit prooxidative activity.^[43]^ In a study by Kovacic et al.^[44]^ in which the reduction potentials of antimycobacterial agents in relation to their activity was investigated, they found that clofazimine, among other agents, undergoes redox cycling and that there is a correlation between reduction potential and activity.

**Figure 2:**
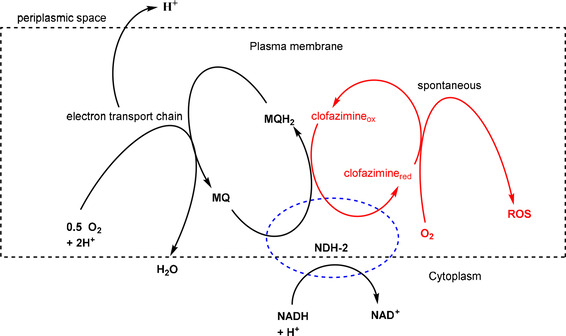
Proposed intracellular mechanism of clofazimine‐mediated redox cycling and ROS production. Menaquinone (MQ) of the respiratory chain and clofazimine are proposed to compete as substrates of NDH‐2. Oxidation of reduced clofazimine by oxygen occurs non‐enzymatically and produces ROS.^[31]^

However, having mentioned all the evidence on the mechanism of action (MOA) on clofazimine upon its discovery, convincing evidence for the existence of such a mechanism was only provided 50 years later by Yano et al.^[31]^ These authors, used isolated membrane fractions from *M. smegmatis* to demonstrate that clofazimine appears to compete for electrons with menaquinone, the substrate for type 2 NADH: quinone oxidoreductase, which is the first step in the mycobacterial respiratory chain (Figure 2). Nonenzymatic oxidation of reduced clofazimine mediated by dissolved intracellular oxygen yields clofazimine and reactive oxygen species (ROS). Its extreme hydrophilicity suggests that it functions in association with membranes to inhibit K+ transportation and DNA binding.^[23]^ Furthermore, menaquinone possesses secondary membrane‐stabilizing properties, which may counteract the disruptive effect of clofazimine on the mycobacterial membrane.^[43]^ Menaquinone of the respiratory chain and clofazimine act as competing substrates of NDH‐2.

**Figure 3:**
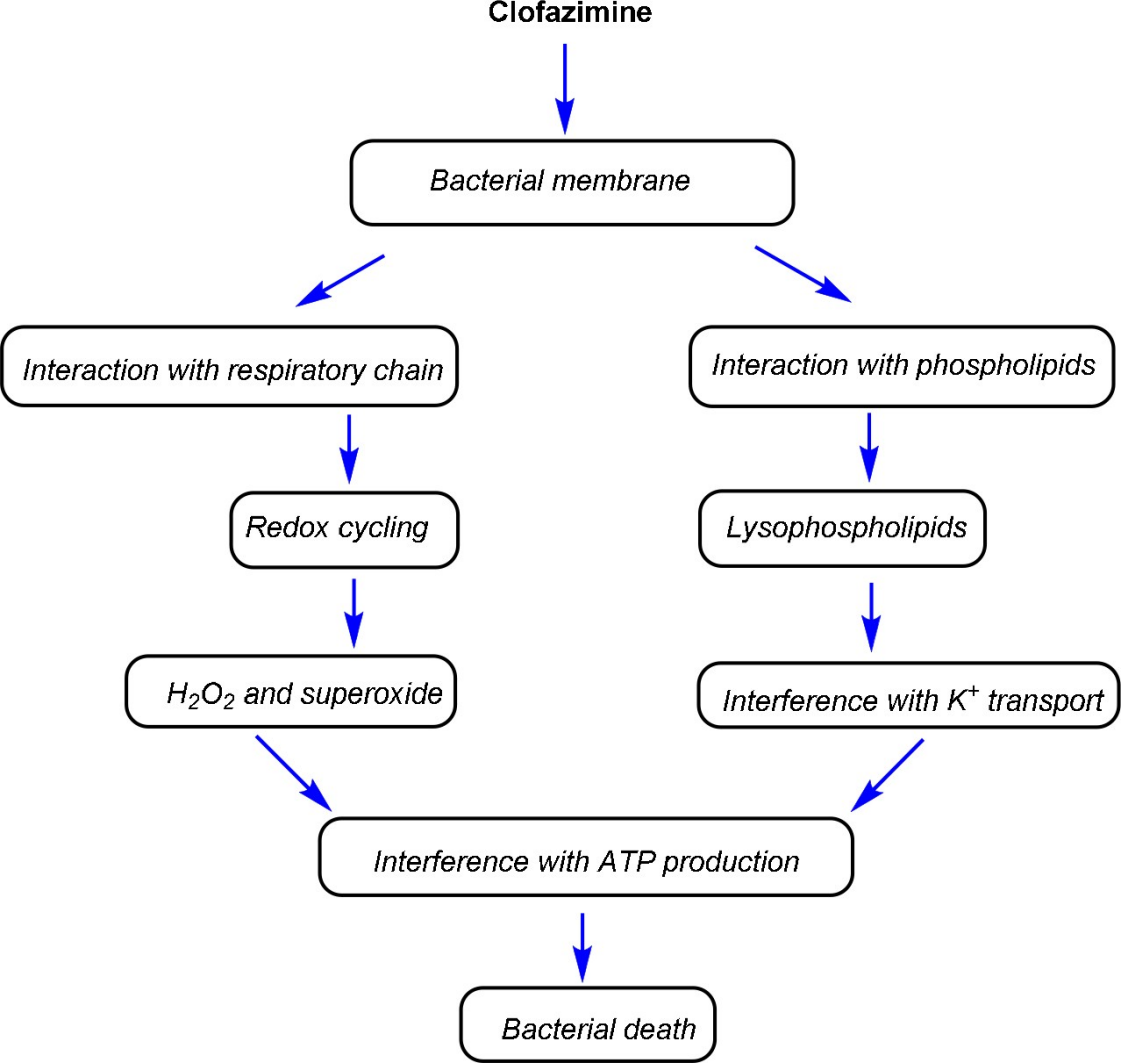
A schematic representation of the mechanism of antimicrobial activity of clofazimine.^[45]^

In addition, Lu et al.^[44]^ reported that the exposure of *M. tuberculosis* to clofazimine at low (<0.16 %) oxygen concentrations leads to moderate loss of antibacterial activity of the antibiotic. The experiment was done using a low oxygen recovery assay (LORA) and the results led the authors to propose that, under different environmental conditions, clofazimine‐mediated antimycobacterial mechanisms might be operative. Cholo et al.^[45]^ provided insights into the mechanisms of antimicrobial activity of clofazimine, as summarized in Figure 3. These mechanisms are membrane‐directed, targeting the respiratory chain and ion transporters. Intracellular redox cycling which involves oxidation and reduction of clofazimine leads to the generation of antimicrobial ROS, superoxide and H_2_O_2_. Secondly, the interaction of clofazimine with membrane phospholipids and then results in the generation of antimicrobial lysophospholipids, which promote membrane dysfunction, causing interference with the uptake of K^+^. Both these mechanisms result in the interference with cellular energy metabolism and consequently lead to the death of the bacteria.^[45]^

### 2.3 Physicochemical properties

In general, antibacterial drugs possess unique physicochemical properties that are different from other drugs. Due to the unique design of bacterial cell walls, specific physicochemical features are required which enhance the permeability of drug molecules across the cell membrane. Properties that contribute to the uniqueness of these drugs include lower lipophilicities, higher molecular weights and increased total polar surface area when compared to drugs for human hosts in other therapies.^[46]^

One physicochemical property of great importance for riminophenazines is lipophilicity (C log *P*). Clofazimine is known to have high lipophilicity, and therefore, accumulates extensively in skin and fat tissues, leading to an extremely long terminal half‐life (*t*_1/2_; >70 days in humans) and undesirable side eﬀects such as skin discolouration.[^47^, ^48^, ^49^] However, most reported riminophenazine derivatives have lower C log *P* compared to that of clofazimine.[^50^, ^51^, ^52^] *M. tuberculosis* cells have an extremely lipophilic cell wall and, therefore, require lipophilic molecules to penetrate through passive uptake.

## 3 Antituberculosis Activity of Riminophenazines

Clofazimine, also known as B663, is one of the most researched riminophenazine compounds. Its minimum inhibition concentration (MIC) value for *M. tuberculosis* strains ranged from 1.3 mg/L to 3.3 mg/L in Proskauer and Beck medium.^[28]^ Its *in vivo* activity was investigated in various animal studies including mice, guinea pigs, rabbits, hamsters and monkeys. The highest tuberculosis activity was seen in the mice, with good activities in hamsters and rabbits. The drug was, however, less effective in guinea pigs and monkeys.^[26]^

Since the discovery of clofazimine, hundreds of its analogues or riminophenazine compounds have been synthesized and tested.[^32^, ^33^, ^34^, ^36^, ^37^, ^38^, ^39^, ^40^, ^53^] In the 1980s and 1990s, the focus from most studies was on the activities against different mycobacteria such as *M. leprae*, *M. avium complex*, *M. smegmatis* with only two studies looking at *M. tuberculosis*.[^53^, ^54^]

**Figure 4:**
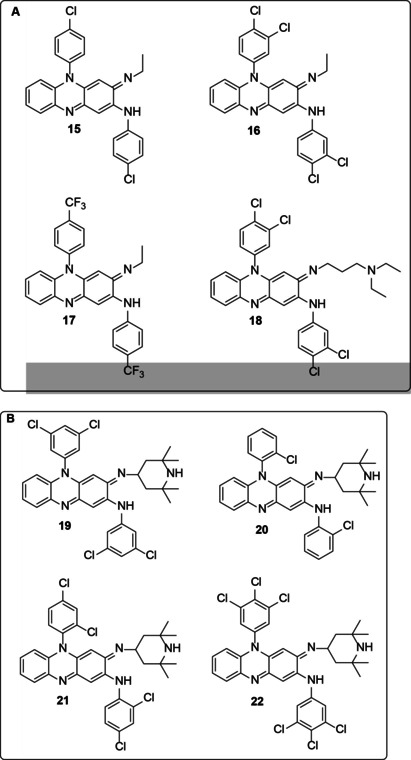
A) Some of the tested compounds against *Mycobacterium Tuberculosis* B) The tetramethylpiperidine (TMP)‐substituted phenazines.

Although many more compounds were synthesized, only a few were selected based on their different outcomes (e. g., intracellular activity) for antibacterial activity (Figure 4A). Compounds **15**, **16**, **17** and **18** were shown to be superior to clofazimine in *in vitro* testing. When used in animals, compounds **15**, **17** and **18** showed less pigmentation than either isoniazid or rifampicin as control drugs.[^53^, ^54^]

Any skin discolouration due to a drug is a cause of great concern. For clofazimine, its high lipophilicity results in accumulation and discolouration. The tendency for accumulation and crystallization within fatty tissues and the tissues of the mononuclear phagocyte system (MPS) causes gastrointestinal and ocular harm, hence leading to unwelcome skin discolouration.

Therefore, one of the key goals of drug research is to reduce the occurrence of discolouration.

In another study,^[55]^ researchers looked at four new synthesized tetramethylpiperidine (TMP)‐substituted phenazines (**19**, **20**, **21** and **22**; Figure 4B) and compared their intra‐ and extracellular activities against *M. tuberculosis* H_37_Rv (ATCC 27294) to those of clofazimine and rifampicin. MIC values ranging from 0.015 μg/mL to 0.03 μg/mL were observed for **19** and **22** against *M. tuberculosis* H_37_Rv strain compared to 0.06 μg/mL for clofazimine. Also, in all the concentrations tested (0.001–0.5 μg/mL), synthesized riminophenazine compounds were found to be more impressively active intracellularly than rifampicin, while clofazimine was the least active of the all the riminophenazines. Two of the TMP‐substituted phenazines (**19** and **21**) showed significant intracellular activity in human monocytes/ macrophages (between 45 and 60 % inhibition of growth) against *M. tuberculosis* at a concentration of 0.001 μg/mL. Overall, the TMP‐substituted phenazines had an extra‐ and intracellular anti‐mycobacterial activity which was superior to that of clofazimine. Their efficacy and remarkable intracellular activity against the pathogen made them contenders for more thorough evaluation in murine models of experimental antituberculosis chemotherapy.

**Figure 5:**
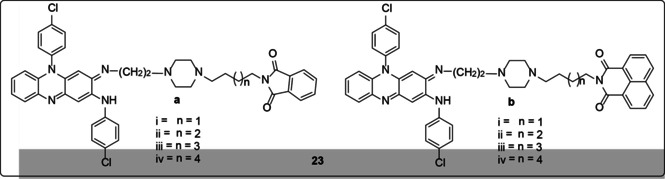
Structures of phthalimido and naphthalimido moieties.

Kamal et al.^[56]^ designed and synthesized new phenazines (**23**) with phthalimido and naphthalimido moieties (Figure 5) and evaluated them for their *in vitro* antitubercular activity against *M. tuberculosis* H_37_Rv ATCC 27294. Compounds **23a_iii_** and **23a_iv_** were the most active compounds of them all. They exhibited MIC values of 1.0 μg/mL against drug‐sensitive strains of *M. tuberculosis* and 1.0–4.0 μg/mL against drug‐resistant clinical isolates of *M. tuberculosis*. The activity of these two compounds was better than that of the standard drug isoniazid on drug‐resistant isolates of *M. tuberculosis*. The findings, therefore, showed that phthalimide‐phenazine conjugates that are linked through piperazine moiety with alkane spacers exhibit good antibacterial activity. Furthermore, it was shown that their activity is enhanced by the increased alkane spacer between the piperazine and phthalimide moieties from 5 to 6 carbons.

**Figure 6:**
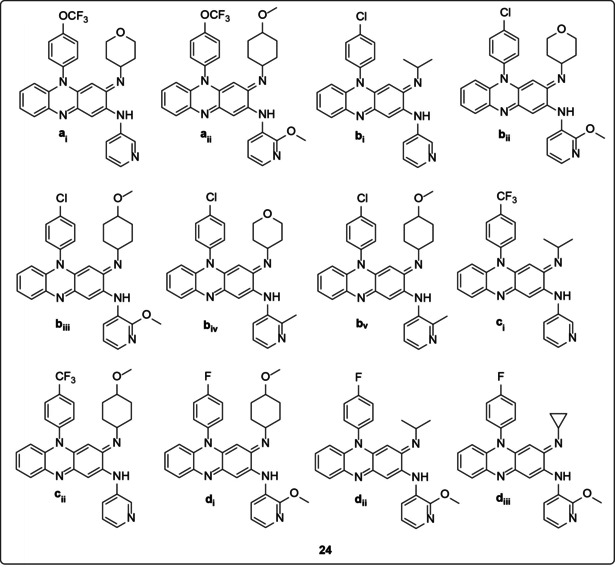
Twelve prioritized riminophenazine compounds.

Other researchers who looked at the potential of riminophenazines for antitubercular therapy investigated the structure‐activity relationship of hundreds of new clofazimine analogues for anti *M. tuberculosis* activity.^[44]^ However, only 12 prioritized compounds (**24**; Figure 6) were reported for different pharmacological properties including lipophilicity, *in vitro* activity against *M. tuberculosis* H_37_Rv, cytotoxicity and selectivity index (SI). All 12 compounds (**24a_i_**–**24d_iii_**) showed excellent activity compared to clofazimine with MIC values ranging from 0.011–0.038 μg/mL. Moreover, the compounds had acceptable cytotoxicity against VERO cells with 7 compound (**24a_i_**, **24a_ii_**, **24b_ii_**, **24b_iii_**, **24d_i_**, **24d_ii_** and **24d_iii_**) all exhibiting IC_50_ values greater than 64 μg/mL prompting the selectivity index of more than that of clofazimine's 572. Also, all the analogues exhibited lower log *P* values (less lipophilicity) than clofazimine (log *P*=5.24). Activity against drug‐sensitive *M. tuberculosis* isolates demonstrated MIC values ranging from 0.015–0.116 μg/mL. Likewise, for the drug‐resistant isolates, the compounds showed improved activities (MIC=0.015–0.121 μg/mL) compared to 0.108–0.24 μg/mL for clofazimine. The selected nine compounds (**24a_ii_**, **24b_i_**, **24b_ii_**, **24b_iii_**, **24b_iv_**, **24b_v_**, **24d_i_**, **24d_ii_**, **24d_iii_**) tested for *in vivo* activity showed better potency with Log10 CFU/lung of 3.64‐4.86 vs 6.33 of clofazimine. In the model presented, all the riminophenazine analogs demonstrated enhanced efficacy compared to clofazimine when administered for 20 days at 20 mg/kg in an acute murine TB model. However, simple inspection of the *in vivo* activities of the compounds compared to their AUC/MIC ratios revealed no facile correlation between the two.

In another structure‐activity relationship study,^[50]^ a number of clofazimine analogues was synthesized and evaluated for their antituberculosis activity (**25**). The study showed that the central tricyclic phenazine nucleus and the pendant aromatic rings are important for antituberculosis activity. This was concluded when compound **25 i** (a‐ring deletion compound) exhibited considerably reduced *in vitro* activity against *M. tuberculosis* (MIC_90_=18.89 μM). Furthermore, when the pendant phenyl groups at N‐5 and C‐2 positions were replaced by a methyl group (compounds **25 ii** and **25 iii**), the antimycobacterial activity was also eliminated (MIC_90_≥42.45 and=21.23 μM, respectively).

**Figure 7:**
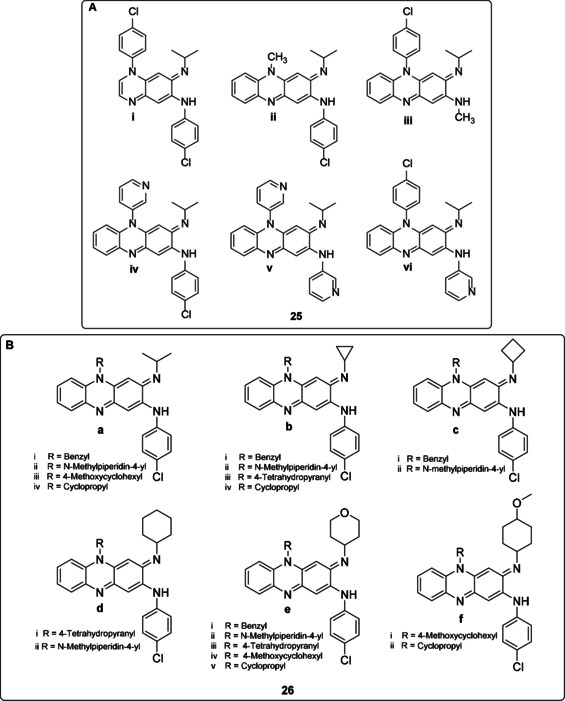
A) Varied symmetrical and asymmetrical riminophenazine compounds B) Riminophenazine compounds with aliphatic N‐5 substituents.

Of all the synthesized compounds illustrated in Figure 7A, **25 vi** demonstrated improved potency against *M. tuberculosis* H_37_Rv with MIC_90_ value of 0.07 μM compared to clofazimine's 0.25 μM. Also, it showed a lower lipophilicity (log *P* of 3.43 vs 5.43 of clofazimine) and excellent *in vivo* efficacy. This was evaluated in a mouse tuberculosis model which showed that the mean log CFU count in the lung dropped by 5 units to the untreated group and by 3 to clofazimine. The study also discovered that the replacement of phenyl group with pyridyl group to the C‐2 position (**25 v**, **25 vi**) of the phenazine ring led to compounds with improved *in vitro* and *in vivo* antituberculosis activity and also favourable pharmacokinetics profiles with reduced skin pigmentation potential.

In continuing efforts, researchers also synthesized 19 riminophenazine compounds (**26**) by introducing aliphatic substituents at N‐5 and C‐3 positions of the phenazine ring (Figure 7B). The substitution of phenyl groups by aliphatic substituents was the first reported by Li et al.^[57]^ The compounds were screened for i) their antitubercular activity against *M. tuberculosis* H_37_Rv strain, ii) for mammalian cytotoxicity using VERO cells line and iii) their lipophilicity. The majority of the compounds were found to be active in inhibiting the *M. tuberculosis*, except for compounds **26e_iii_** and **26a_iii_** with MIC values of 0.959 and 2.651 μg/mL respectively. However, all the compounds (with the exception of **26a_iii_**) had unacceptable cytotoxicity against VERO cells with IC_50_ values of less than 64 μg/mL. Elsewhere, the exhibition of reduced lipophilicity by substitution with 4‐tetrahydropyranyl group at the imino nitrogen, C‐3 position **26e_iii_** was considered an impressive result. Four compounds (**26a_iv_**, **26b_iv_**, **26e_v_** and **26f_ii_**), all with a cyclopropyl substituent at N‐5 position of the ring, demonstrated improved anti‐TB activity (MIC values of 0.096, 0.050, 0.110 and 0.045 μg/mL respectively). It is important to note that **26f_ii_** was shown to be the most potent riminophenazine compound with lower lipophilicity and larger SI ratio making it the valuable new lead for structural modification.

**Figure 8:**
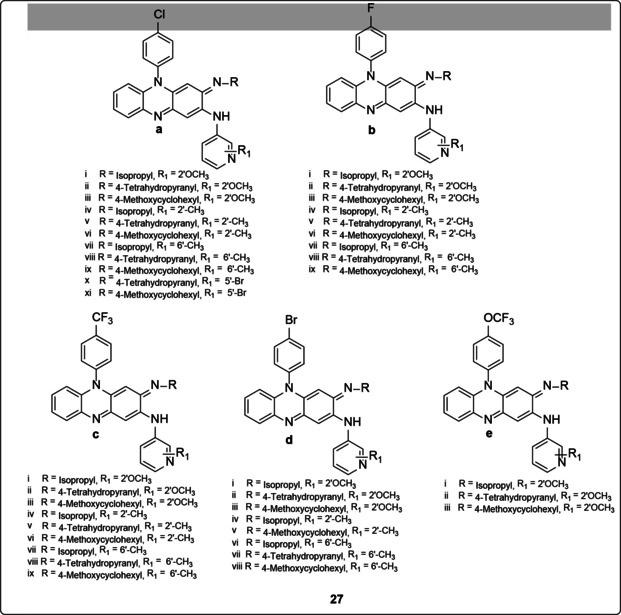
Different asymmetrical riminophenazine compounds.

A series of 69 novel riminophenazine analogues (with only 40 presented in this article, **27**, in Figure 8) which had C‐2 pyridyl substituent were designed, synthesized and evaluated for their *in vitro* activity and cytotoxicity. Also, selected compounds were tested for *in vivo* efficacy against MDR‐TB in the mouse tuberculosis model. Most of the compounds in the series showed lower lipophilicity properties and potent *in vitro* activity against *M. tuberculosis*, with MIC values that ranged from from 0.016–0.125 μg/mL. The substitution, at the C‐3 imino nitrogen, with 4‐tetrahedropyranyl and 4‐methoxycyclohexyl exhibited potent anti‐mycobacterial activity and excellent pharmacokinetic properties. Similarly, substituted pyridyl groups at the C‐2 position of the ring demonstrated potent activity against *M. tuberculosis*. More importantly, many compounds in the series had low cytotoxicity, with IC_50_ values greater than 64 μg/mL. Of the 16 compounds (**27a_ii_**, **27a_iii_**, **27a_vi_**, **27a_ix_**, **27b_i_**, **27b_iii_**, **27b_v_**, **27b_vi_**, **27c_i_**, **27c_ii_**, **27c_iii_**, **27c_v_**, **27d_ii_**, **27d_iii_**, **27d_v_**, **27e_iii_**) selected for the preliminary toxicity study on a single oral dose in mice at 500 mg/kg, 11 of them (**27a_ii_**, **27a_iii_**, **27a_ix_**, **27b_i_**, **27b_iii_**, **27c_i_**, **27c_ii_**, **27c_iii_**, **27d_ii_**, **27d_iii_**, **27e_iii_**) showed 6/6 number of animals that survived. However, six (6) of them (**27a_ii_**, **27a_iii_**, **27a_ix_**, **27b_i_**, **27b_iii_**, **27e_iii_**) were evaluated for MDR‐TB infection in mouse model. They all showed significant efficacy by producing 3–5 logs of CFU reduction in the lungs after 20 days of treatment as compared to untreated mice. Overall, two compounds (**27a_iii_**, **27a_ix_**) showed much improved *in vivo* efficacy against MDR‐TB with reduced discoloration potential in fat tissues and organs when compared to clofazimine.^[51]^

**Figure 9:**
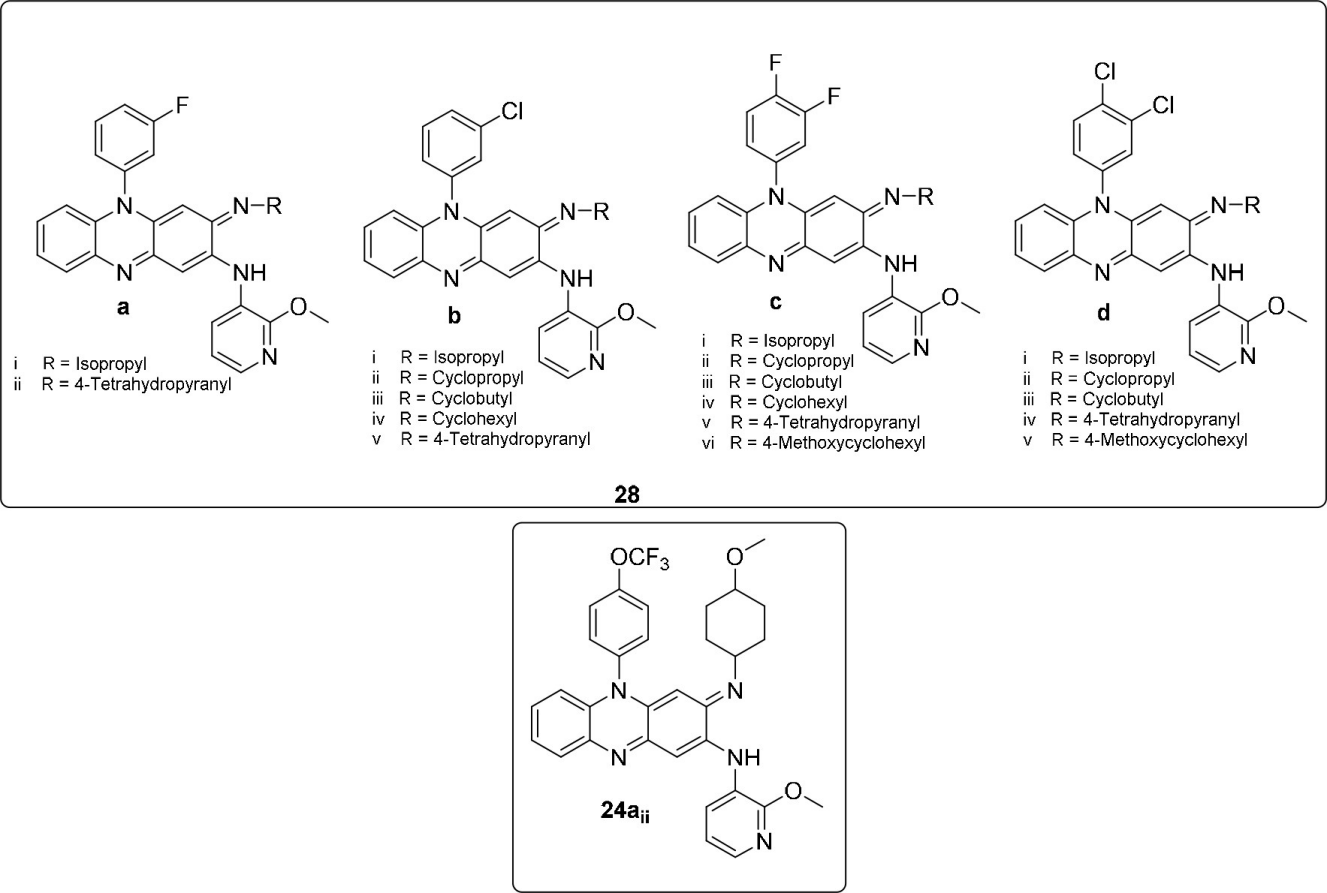
Compounds with 2‐methoxypyridyl substituent at the C‐2 position and structure of compound **24a_ii_** (better known as TBI‐166).

Following their successful study using pyridyl as a substituent at the C‐2 position of the phenazine ring, Zhang et al.^[52]^ went on to identify novel riminophenazine derivatives with potent antituberculosis activity, and improved pharmacokinetic and physicochemical properties. Eighteen novel riminophenazine derivatives (**28**; Figure 9) containing 2‐methoxypyridyl substituent at the C‐2 position of the phenazine ring were synthesized and evaluated for antituberculosis activity against *M. tuberculosis* H_37_Hv and were also screened for cytotoxicity. All derivatives, with the exception of **28b_iv_** showed C log *P* values lower than clofazimine (4.70–7.17). Generally, compounds with 3‐flouro‐ and 3,4‐difluorophenyl‐substituted (**28 a** and **28 c**) groups had reduced lipophilicity when compared to the chloro‐substituted groups (**28 b** and **28 d**). Against *M. tuberculosis* H_37_Rv, it appeared that the position and the type of the halogen atoms on the phenyl ring at N‐5 position had no significant impact on the activity of the bacteria. The majority of the compounds showed potent activity ranging from 0.01–0.1 μg/mL. However, on the cytotoxicity, the halogen substitution pattern appears to be important. This was seen with the 3,4‐dihalogen substitution pattern which displayed significantly lower cytotoxicity hence higher SI values. Compounds **28c_i_** and **28d_ii_** were selected for multiple doses in *in vivo* toxicity studies in mice, and they both exhibited tolerability in mice. In particular, **28c_i_** demonstrated excellent efficacy in mice infected with *M. tuberculosis* H_37_Rv with the bacterial burden in the lungs reduced to 3.8 logs CFU. Furthermore, it also demonstrated shorter plasma half‐life (1/2), higher *C*_max_ and AUC, suggesting potential as a new drug candidate for the treatment of TB and MDR‐TB.

Very recently, researchers^[58]^ investigated intensively the *in vitro* and *in vivo* activities of compound **24a_ii_** (Figure 9; better known as TBI‐166 from a previous study by Lu et al.^[44]^). In the original study, the compound demonstrated enhanced *in vitro* activity compared to clofazimine against replicating *M. tuberculosis* H_37_Rv with MIC value of 0.016 μg/mL. Dosed at 20 mg/kg of body weight/day for 3 weeks, TBI‐166 showed an efficacy (i. e., CFU reduction) superior to that of 20 mg/kg clofazimine in an acute murine TB model. The compound also showed improved lipophilic properties with reduced lipophilicity (log *P* 4.52). This was attributed to the replacement of the C‐2 phenyl ring in clofazimine with 2‐methoxypyridyl group, suggesting that TBI‐166 may decrease the potential for tissue discolouration.

The study also investigated the compound's *in vitro* activity against *M. tuberculosis* clinical isolates and other mycobacterial species, as well as its efficacy against TB in murine models. The compound's MIC value against replicating *M. tuberculosis* H_37_Hv was 0.063 μg/mL. When tested against 16 drug‐sensitive clinical isolates, it ranged from <0.005–0.15 μg/mL whilst against 28 drug‐resistant clinical isolates it ranged between 0.01–0.02 μg/mL. All these values were lower compared to clofazimine.

For the acute infection model, the compound showed mean lung CFU counts for all doses to be lower than those of the untreated control group, whereas against chronic murine TB infected with *M. tuberculosis* H_37_Hv, it demonstrated time‐dependent killing of the bacteria with lung CFU decline at 8 weeks. The induced discolouration of the compound was observed in the ears and fat of mice during the 8 weeks of treatment. Elsewhere, the compound showed a shorter plasma half‐life of about 45 h compared to clofazimine's 65 h in a single‐dose pharmacokinetics study. Overall, the compound's concentrations were higher in fat, skin, lungs, liver and spleen, though it exhibited reduced discolouration of adipose tissue and skin compared to clofazimine. Following preliminary evaluations of pharmacokinetics and extensive evaluations of efficacy, TBI‐166 was selected as a preclinical development candidate and is therefore under development in a phase I clinical trial in China. The compound has been documented by the name pyrifazimine and its MOA has been found to inhibit ion transport and bacterial respiration.[^58^, ^59^]

In another study,^[60]^ TBI‐166 was assessed for pharmacological interactions *in vitro* and *in vivo* with several anti‐TB drugs, including isoniazid (INH), rifampin (RFP), bedaquiline (BDQ), pretomanid (PMD), linezolid (LZD), and pyrazinamide (PZA). It was found that in BALB/c mice, five TBI‐166‐containing regimens showed significantly more potent efficacy after 4 weeks of treatment compared to the control regimen, INH+RFP+PZA with the rank order of the potency being as follows: TBI‐166+BDQ+LZD>TBI‐166+BDQ>TBI‐166+PZA>TBI‐166+BDQ+PMD+LZD>TBI‐166+BDQ+PMD. As a results, the TBI‐166+BDQ+LZD regimen is recommended for further testing in a TBI‐166 phase IIb clinical trial.

## 4 Summary and Outlook

Basic drug resistance remains the main challenge to achieving effective results with antituberculosis drugs and this is a major public health problem with a serious global impact.^[19]^ The discovery of new antituberculosis drugs to treat TB is, therefore, necessary. It is evident from the above discussion that riminophenazines/clofazimine derivatives possess enormous potential as antituberculosis agents. These derivatives have attracted a lot of attention from researchers.

This minireview focused on the antituberculosis activity of recently developed riminophenazines/clofazimine derivatives. In general, almost all of the riminophenazines which were reviewed in this article appear to be potent in both *in vitro* and *in vivo* activity against the *M. tuberculosis* H_37_Hv strain.

**Figure 10:**
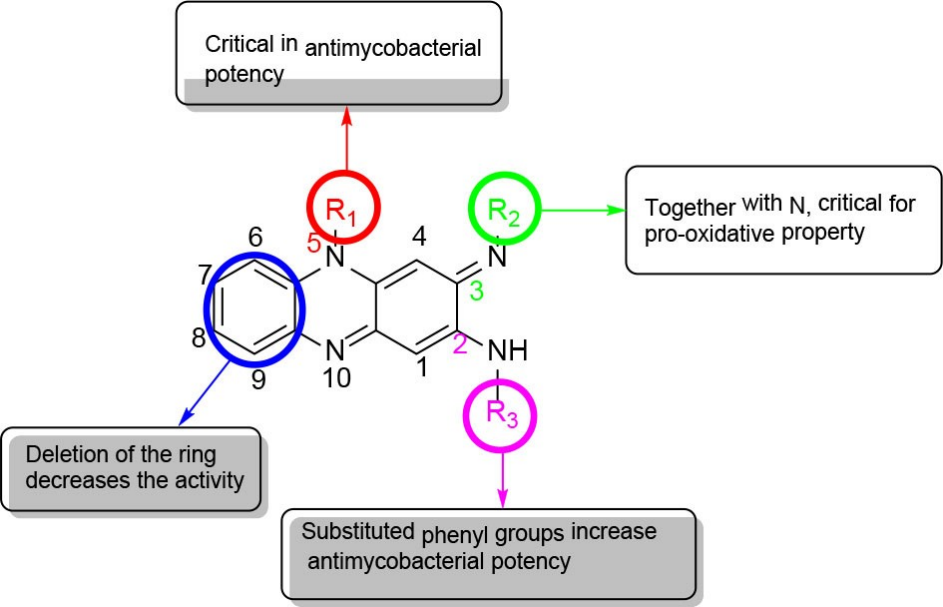
Structural overview of SAR study.

An analysis of their structural (Figure 10) features showed that:

- the three rings of the phenazine nucleus are believed to be the pharmacophore of the molecules and hence beneficial towards their biological activities. This was shown by the deletion of a ring compound **25 i** which reduced its *in vitro* activity.
- the substitution of the phenyl group at N‐5 position of the phenazine nucleus with aliphatic groups **26** demonstrated improved physicochemical properties and biological activities. These included potent antitubercular activity against the *M. tuberculosis* H_37_Rv strain, good cytotoxicity levels hence higher selectivity index (SI) values, and finally reduced lipophilicity.
- replacement of the phenyl group with pyridyl at the C‐2 (**24**, **27**, **28**) position yielded excellent biological properties. Both *in vitro* (potent antituberculosis action against *M. tuberculosis* H_37_Hv strain) and *in vivo* assays (exhibited equal or better eﬃcacy against MDR‐TB than clofazimine with reduced discoloration potential in fat tissues and organs) displayed exceptional results.

In general, fine‐tuning the substituents on the phenazine backbone may afford novel molecules with enhanced antituberculosis potency, as well as drug‐like properties. Therefore, development of more promising analogues such as TBI‐166 or **24b_ii_** (with pyridyl substituent at the C‐2 position) which has since progressed to clinical phase I trials in China and along with other drugs is endorsed for clinical phase II trials should be encouraging to scientists for further research pursuits in this direction. This review article provided good indications and a broader scope for further investigation towards the design and development of riminophenazines in tuberculosis drug research.

## Conflict of interest

The author wishes to declare no conflict of interest.

## Biographical Information

*Mpelegeng Victoria Bvumbi graduated with an Honours degree in chemistry (2007) and obtained her MSc in biophysical and structural chemistry (2009) from the University of Cape Town. As a student researcher at the Council of Science and Industrial Research (CSIR), she synthesized and conducted the biological evaluation of phenazines targeting redox cascades in tuberculosis. This led to her obtaining her PhD in medicinal chemistry from the University of Johannesburg in 2017. After a brief stint as a postdoctoral fellow at the University of Venda, working on several medicinal chemistry projects, she is currently employed as a Chemistry Lecturer in a contractual position at the same institution*.